# A *Sargassum fusiforme*-Derived Fucoidan Preparation Ameliorates Loperamide-Induced Constipation and Is Associated with Changes in Colonic Inflammation and Gut Microbiota in Mice

**DOI:** 10.3390/md24080266

**Published:** 2026-07-31

**Authors:** Jun-Geon Je, Chan-Young Kim, Sang-Woon Lee, Rajasinghe Peli Gedara Sewwandi Kaushalya Amarasiri, Jimin Hyun, Bomi Ryu, You-Jin Jeon

**Affiliations:** 1 Department of Marine Life Sciences, Jeju National University, Jeju 63243, Republic of Korea; wpwnsrjs@gmail.com (J.-G.J.); kimsline01@naver.com (C.-Y.K.); pucu2195@gmail.com (S.-W.L.); sewwandi1295@gmail.com (R.P.G.S.K.A.); 2 Major of Food Science and Nutrition, Pukyong National University, Busan 48513, Republic of Korea; localman1727@gmail.com (J.H.); bmryu@pknu.ac.kr (B.R.)

**Keywords:** *Sargassum fusiforme*, fucoidan, constipation, MAPK, gut microbiota

## Abstract

Chronic constipation is a prevalent functional gastrointestinal disorder with limited long-term treatment options. This study examined whether a fucoidan preparation derived from *Sargassum fusiforme* (SF) ameliorates loperamide (LOP)-induced constipation in male ICR mice. SF was administered by oral gavage at 50, 100 or 200 mg/kg/day for 35 days, and constipation was induced with LOP (5 mg/kg) during the final 7 days; the design is therefore preventive rather than therapeutic. Defecation endpoints were recorded at the cage level (*n* = 3 cages per group) and fecal moisture in individual mice (*n* = 7 per group), whereas colonic cytokine and myeloperoxidase (MPO) measurements (*n* = 3–4 per group) and Western blot analyses (*n* = 3 per group) were performed in a small subset of animals, and histological analyses in *n* = 6–8 per group. SF increased fecal output and fecal moisture content with increasing dose; at 200 mg/kg the relative fecal number returned to the level of the Vehicle control group, although the absolute daily pellet count remained lower than that of the Vehicle control and of the Positive control group. SF administration was associated with lower colonic TNF-α, IL-6, IL-1β and MPO concentrations, with reduced phosphorylation of p38, JNK and ERK, with higher C-kit and stem cell factor (SCF) levels, with normalization of the LOP-induced increase in aquaporin-3 (AQP3), and with recovery of colonic mucosal thickness and mucin content. 16S rRNA gene sequencing showed a shift in fecal microbiota community structure (PERMANOVA *p* = 0.0002) and a dose-related increase in the combined relative abundance of Lactobacillus and Bifidobacterium, without a reduction in alpha diversity. These findings indicate that SF ameliorates LOP-induced constipation in male mice and that this effect is accompanied by changes in colonic inflammatory signaling, motility-related protein expression, mucosal architecture and gut microbiota composition.

## 1. Introduction

Gut health is increasingly recognized as relevant to systemic physiology [1]. The gut microbiota has been associated with host immune regulation [2], with weight and metabolic regulation, and with skin homeostasis [3,4]. Dietary patterns [5], psychosocial stress and antibiotic exposure can alter microbial community composition, with downstream consequences for gut–brain signaling [6,7].

Stool frequency and stool consistency are widely used clinical indicators of colonic transit and are among the domains covered by current consensus definitions of gut health [8]. They also form part of the diagnostic criteria for chronic constipation [9]. As one of the most prevalent functional gastrointestinal disorders, chronic constipation significantly impairs quality of life by causing physical distress that frequently escalates into psychological burden, social embarrassment, and diminished overall life satisfaction [10,11]. Chronic constipation affects approximately 14–16% of the global population [12,13], and its incidence continues to rise in parallel with Westernized dietary habits, sedentary lifestyles, and an aging global population [14]. First-line pharmacological management of chronic idiopathic constipation relies on osmotic and stimulant laxatives, which current clinical practice guidelines regard as effective and generally well tolerated [15]. Nevertheless, a substantial proportion of patients report dissatisfaction with available therapies, and adverse effects such as abdominal cramping and bloating limit tolerability in some individuals [16]. This has sustained interest in dietary approaches to supporting bowel regularity.

Emerging evidence has reframed constipation as a multifactorial condition closely linked to colonic inflammation, impaired intestinal motility, mucosal barrier deterioration, and gut microbial dysbiosis [17,18]. The p38, JNK and ERK MAPK pathways are established regulators of pro-inflammatory cytokine production in intestinal tissue, although this evidence derives largely from inflammatory bowel disease [19,20]. Their contribution to constipation is less well characterized, and we therefore examined MAPK phosphorylation as an exploratory indicator of colonic inflammatory signaling. The interstitial cells of Cajal (ICC), which function as intestinal pacemakers and are characterized by the expression of C-kit and its ligand stem cell factor (SCF), are markedly diminished under constipation conditions, compromising the coordinated peristaltic contractions necessary for normal transit [21,22]. Aquaporin-3 (AQP3) is a water channel expressed in the colonic epithelium, and altered colonic AQP3 expression has been reported in constipation models [23]. Because AQP3 protein abundance does not by itself establish a change in transepithelial water flux, AQP3 expression is interpreted here as an associated molecular change rather than as direct evidence of altered water absorption. Thinning of the colonic mucosa and depletion of goblet cell-derived mucin have been reported in functional constipation [24] and are used as histological indicators of impaired mucosal integrity [25]. At the ecosystem level, reduced abundance of beneficial genera such as Lactobacillus and Bifidobacterium and an overall shift in community composition further perpetuate the constipatory phenotype [26]. Collectively, these interconnected pathological axes —inflammatory signaling, motility impairment, mucosal deterioration, and microbial imbalance—highlight the necessity of therapeutic agents capable of simultaneously addressing multiple targets.

Marine macroalgae have garnered considerable scientific attention as sources of structurally diverse bioactive polysaccharides with broad health-promoting potential [27]. Among brown algae, *Sargassum fusiforme*, a seaweed consumed for centuries across East Asian countries including Korea and Japan is particularly distinguished by its high content of fucoidan, a sulfated fucose-rich polysaccharide [28]. The fucoidan derived from *S. fusiforme* is characterized by an elevated degree of sulfation relative to fucoidans from other brown algal species, a structural feature recognized as a key determinant of its potent anti-inflammatory and immunomodulatory properties [29,30]. As a soluble dietary fiber and prebiotic substrate, SF fucoidan supports the proliferation of beneficial gut bacteria and contributes to the maintenance of intestinal homeostasis [31]. Crucially, *S. fusiforme* possesses high regenerative capacity and is cultivable in large quantities as a sustainable marine resource, rendering it an industrially viable candidate for functional food development [32].

The biological activities of *S. fusiforme*-derived fucoidan (SF) have been documented across several pathological contexts. Anti-inflammatory, antioxidant, and immunomodulatory effects have been reported in vitro and in vivo [33,34]. Fucoidan preparations from brown seaweeds have been subjected to dedicated oral toxicity evaluation: no adverse effects were observed in rats after four weeks of administration at 1350 mg/kg/day [35], no toxicologically significant changes were detected up to 1000 mg/kg/day over 28 days, with negative Ames and bone-marrow micronucleus assays [36], and a no-observed-adverse-effect level of 2000 mg/kg/day has been reported for a low-molecular-weight fucoidan [37]. The doses used in the present study (50–200 mg/kg/day) are therefore 5- to 40-fold below these no-effect levels. This evidence relates to purified fucoidan preparations from other brown algal species rather than to *S. fusiforme* fucoidan specifically, and no toxicological evaluation was undertaken in the present study. Although *S. fusiforme* byproduct extracts have been reported to improve intestinal motility in a loperamide-induced constipation model [38], the efficacy of standardized, highly sulfated SF in this context—particularly with respect to MAPK-driven colonic inflammation, ICC pacemaker function, AQP3-mediated water homeostasis, mucosal structural integrity, and gut microbiota composition—has not been systematically investigated.

Therefore, the present study was designed to comprehensively evaluate the anti-constipation efficacy of SF using a loperamide (LOP)-induced constipation mouse model. Specifically, we examined whether SF administration at doses of 50, 100, and 200 mg/kg could restore bowel movement, suppress colonic pro-inflammatory cytokine production (TNF-α, IL-6, IL-1β, and MPO) via MAPK pathway inhibition, normalize ICC-related motility markers (C-kit and SCF) and AQP3 expression, preserve colonic mucosal architecture and mucin content, and modulate gut microbiota composition. Our findings characterize the intestinal changes associated with *S. fusiforme*-derived fucoidan administration in this model and indicate that it merits further evaluation as a candidate dietary ingredient for supporting bowel regularity.

## 2. Results

### 2.1. Effect of SF on General Health (Body Weight, Feed and Water Intake)

**Figure 1:**
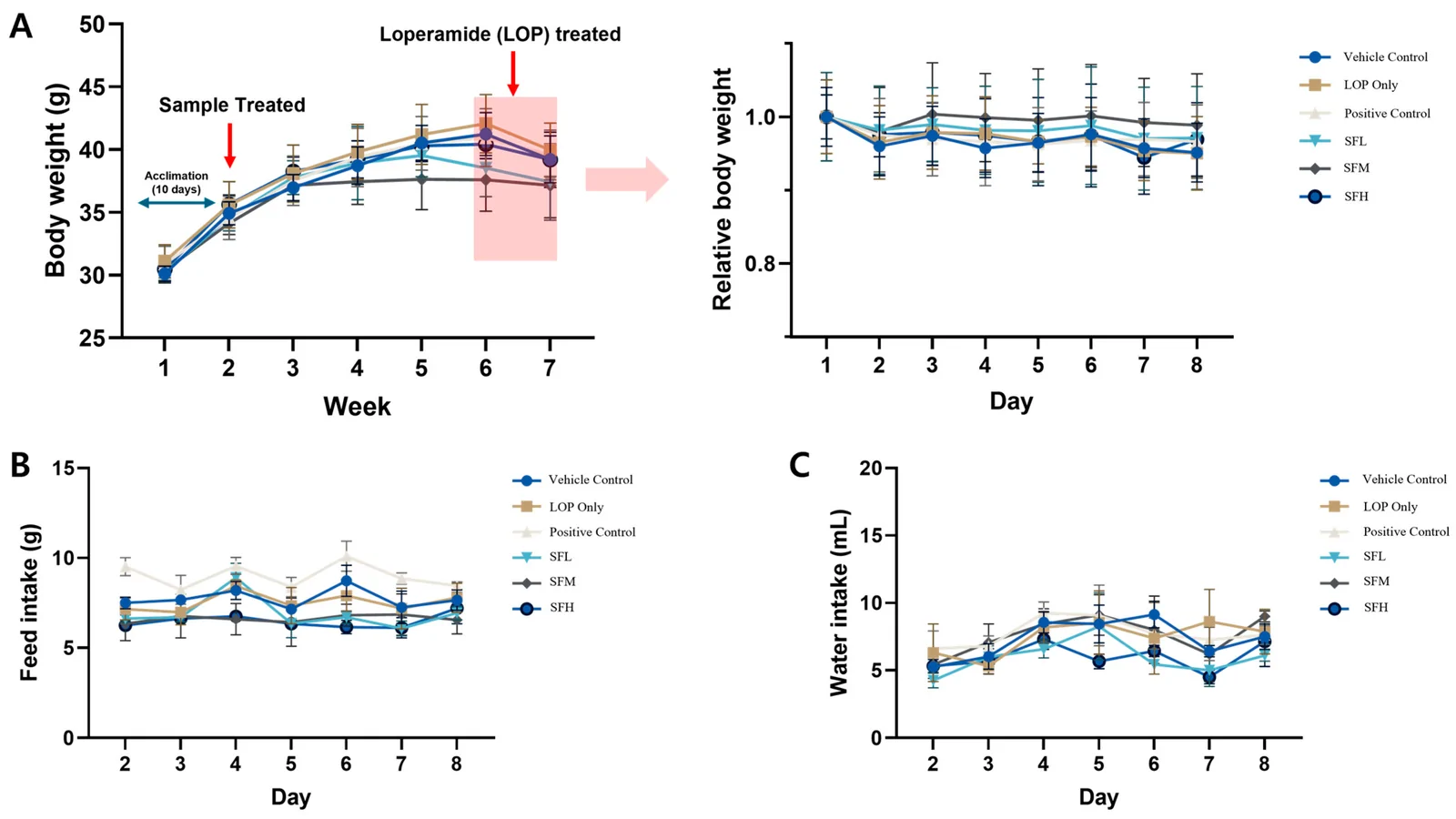
General health during the experimental period. Mice were acclimatized for 10 days (Day −10 to Day 0), received SF by daily oral gavage from Day 1 to Day 35, and were given loperamide (LOP) from Day 29 to Day 35. (**A**) Body weight recorded weekly during the SF administration period and relative body weight during the LOP induction period (Days 29–35, normalized to Day 29), (**B**) feed intake (g) and (**C**) water intake (mL) during the LOP induction period. Data are mean ± SD. For (**A**) the mouse is the experimental unit (*n* = 10–11 mice per group); for (**B**,**C**) feed and water were supplied to and measured from each cage, so the cage is the experimental unit (*n* = 3 cages per group).

Throughout the 5-week SF administration period, body weight increased progressively in all groups, with no significant difference observed between the Vehicle control and SF-treated groups at any time point. Following LOP administration, the LOP only group showed a slight decrease in body weight relative to its pre-LOP value; however, this reduction was not statistically significant (Figure 1). The rate of body-weight change (relative body weight), expressed as a ratio to the value recorded on Day 29 (first day of LOP treatment), likewise did not differ significantly among groups throughout the 8-day LOP induction period. Similarly, neither feed intake nor water intake, measured daily from Day 29 to Day 35, differed significantly between the Vehicle control and any SF-treated group. These results indicate that SF (50–200 mg/kg) had no adverse effect on general health, and that the subsequent improvements in defecation parameters were not attributable to altered food or water consumption.

### 2.2. Effect of SF on Bowel Movement (Fecal Number and Moisture)

**Figure 2:**
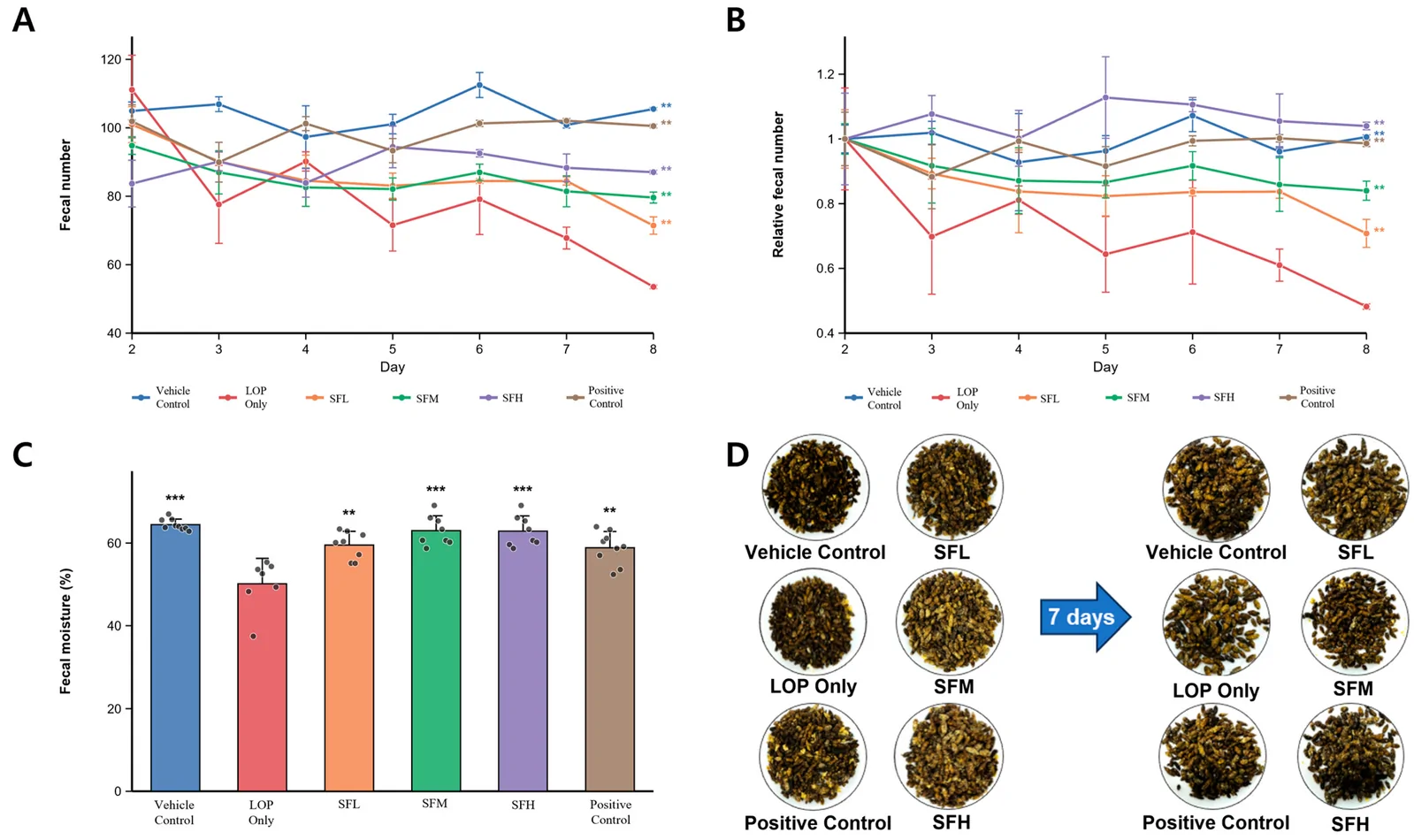
Effect of SF on bowel movement in loperamide-induced constipated mice. (**A**) Daily fecal pellet count and (**B**) relative fecal pellet count, normalized to the Day 29 value, over the loperamide induction period (Days 29–35). Pellets were collected from trays placed beneath group-housed cages, so the cage is the experimental unit for panels: mice were housed in 3 cages per group (3–4 mice per cage), the pellets recovered from each tray were divided by the number of mice housed in that cage, and data are presented as mean ± SD of the 3 cage-level values (*n* = 3 cages per group). Day 29 is the first day of loperamide administration and pellets were collected after dosing, so it does not represent a loperamide-naive baseline. (**C**) Fecal moisture content (%) determined from pellets collected from individually housed mice over a 30-min period on Day 35; for this panel the mouse is the experimental unit and data are presented as individual values with the group mean (*n* = 7 mice per group; mice that did not defecate within the 30-min collection window were not included). (**D**) Representative photographs of the fecal pellets of each group before loperamide induction (Day 29) and after 7 days of loperamide treatment (Day 35); the arrow denotes the interval between the two time points. Comparisons against the LOP only group in (**A**–**C**) were made by Welch’s *t*-test at the level of the corresponding experimental unit. ** *p* < 0.01, *** *p* < 0.001 vs. LOP only.

LOP administration markedly suppressed defecation relative to the Vehicle control group. As shown in Figure 2A, fecal number in the LOP only group declined progressively over the LOP induction period, reaching 53.5 ± 0.5 on Day 35, representing an approximately 49% reduction compared to the Vehicle control group (105.5 ± 0.4; *p* < 0.001). SF supplementation attenuated this decline in a dose-related manner: fecal number on Day 35 was 71.4 ± 2.5, 79.6 ± 1.6, and 87.0 ± 0.6 in the SFL, SFM, and SFH groups, respectively (all *p* < 0.001 vs. LOP only). The relative fecal number, normalized to the Day 29 baseline (Figure 2B), showed the same dose-dependent pattern: the LOP only group declined to 0.48 ± 0.01 by Day 35, whereas SFL, SFM, and SFH recovered to 0.71 ± 0.04, 0.84 ± 0.03, and 1.04 ± 0.01, respectively, with SFH remaining close to the Vehicle control level (Vehicle control: 1.01 ± 0.01; *p* < 0.01 vs. LOP only). Fecal moisture content, measured from pellets collected over a 30-min period on Day 35, was significantly reduced in the LOP only group relative to Vehicle control (50.1 ± 6.2% vs. 64.5 ± 1.4%; Figure 2C). SFL partially recovered moisture (59.7 ± 3.2%; *p* < 0.01 vs. LOP only), while SFM (63.0 ± 3.6%) and SFH (62.9 ± 3.7%) maintained fecal moisture at levels comparable to Vehicle control (both *p* < 0.001 vs. LOP only). On Day 35, SFH and the Positive control group reached similar relative fecal numbers (1.04 ± 0.01 and 0.99 ± 0.01) and similar fecal moisture contents (62.9 ± 3.7% and 58.4 ± 3.7%), whereas the absolute daily pellet count in SFH (87.0 ± 0.6) remained lower than in the Positive control group (100.5 ± 0.6) and than in the Vehicle control group (105.5 ± 0.4) (*p* < 0.01 vs. LOP only). Representative photographs of the pellets are shown in Figure 2D. Before loperamide induction the gross appearance of the feces was comparable among the groups, whereas after 7 days of loperamide treatment the LOP only group produced visibly fewer, smaller, drier and more compact pellets than the Vehicle control group. SF treatment progressively restored both the number and the gross appearance of the pellets, with the SFH group most closely resembling the Vehicle control and Positive control groups. These observations are qualitative and were not quantified.

### 2.3. Effect of SF on Colonic Pro-Inflammatory Cytokines

**Figure 3:**
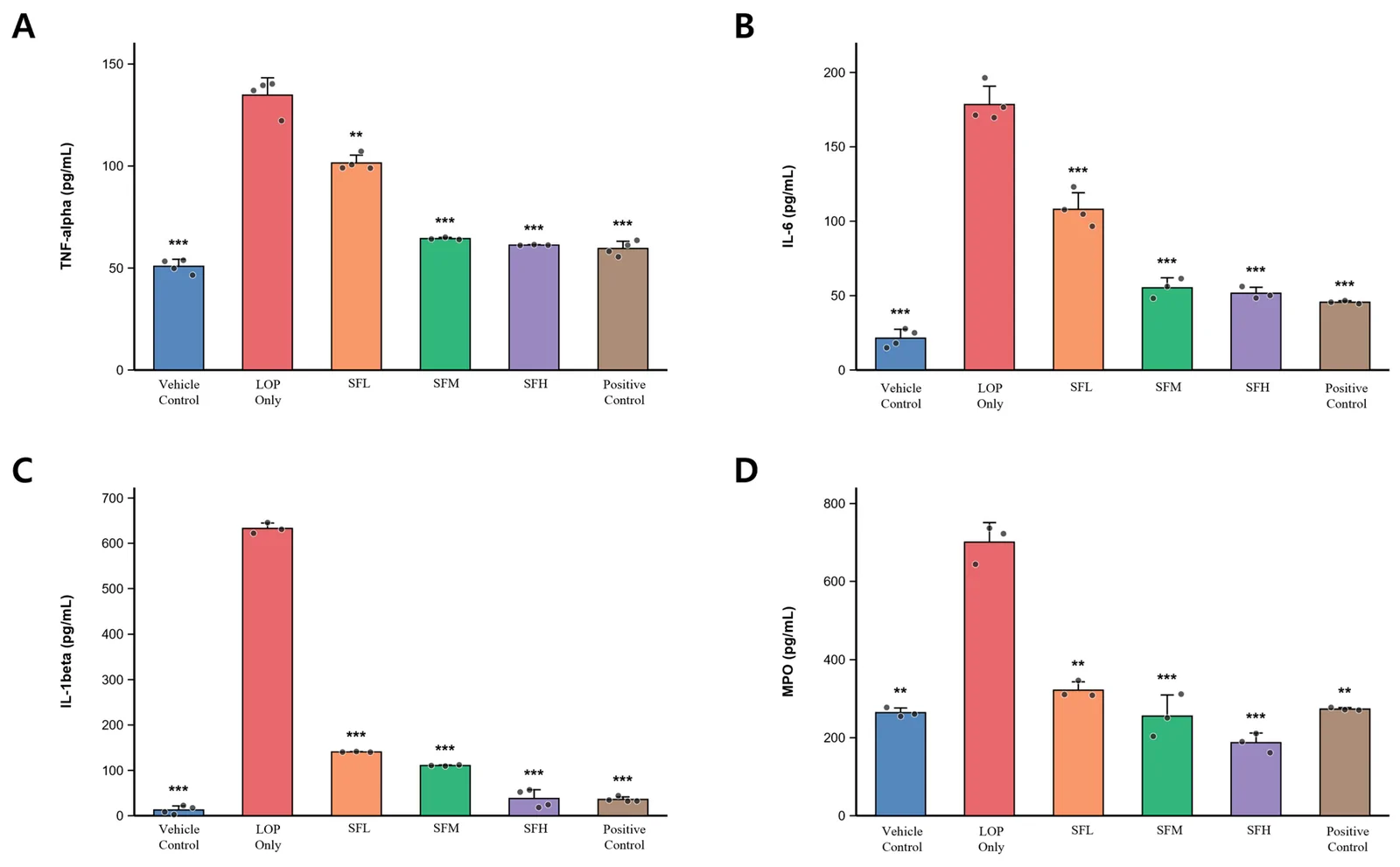
Effect of SF on colonic pro-inflammatory cytokines in loperamide-induced constipated mice. (**A**) TNF-α, (**B**) IL-6, (**C**) IL-1β, and (**D**) myeloperoxidase (MPO) levels in colonic tissue. Data are presented as individual values with mean (*n* = 3–4 per group). ** *p* < 0.01, *** *p* < 0.001 vs. LOP only.

LOP-induced constipation was accompanied by marked colonic inflammation (Figure 3). TNF-α levels were significantly elevated in the LOP only group compared to Vehicle control (134.75 ± 8.49 vs. 50.86 ± 2.96 pg/mL; *p* < 0.001; Figure 3A). SF dose-dependently suppressed TNF-α: SFL 101.49 ± 3.87 pg/mL (*p* < 0.01), SFM 64.43 ± 0.59 pg/mL (*p* < 0.001), and SFH 61.26 ± 0.21 pg/mL (54.5% reduction vs. LOP only; *p* < 0.001), approaching the level of the Positive control group (59.61 ± 3.52 pg/mL). Similarly, IL-6 was markedly elevated in the LOP only group (178.47 ± 12.30 vs. 21.43 ± 5.95 pg/mL in Vehicle control; Figure 3B) and was suppressed by SF in a dose-dependent manner: SFL 108.06 ± 11.09, SFM 55.29 ± 6.67, and SFH 51.58 ± 4.03 pg/mL (71.1% reduction; all *p* < 0.001 vs. LOP only), comparable to bisacodyl (45.55 ± 1.05 pg/mL). IL-1β showed the most dramatic elevation in the LOP only group (632.78 ± 11.95 vs. 12.58 ± 7.78 pg/mL in Vehicle control; Figure 3C), and SF produced the most pronounced suppression at SFH (37.78 ± 19.54 pg/mL; 94.0% reduction vs. LOP only; *p* < 0.001), a value comparable to bisacodyl (35.78 ± 5.63 pg/mL) but still approximately three-fold higher than that of the Vehicle control group (12.58 ± 7.78 pg/mL). MPO, a marker of neutrophil infiltration, was similarly elevated in the LOP only group (700.91 ± 50.13 vs. 264.22 ± 9.76 pg/mL in Vehicle control; Figure 3D) and was suppressed by SFL (321.99 ± 21.34 pg/mL; *p* < 0.01), SFM (255.33 ± 54.14 pg/mL; *p* < 0.001), and SFH (187.25 ± 24.58 pg/mL; 73.3% reduction; *p* < 0.001), with SFH reducing MPO below the Vehicle control level. Across the ordered dose groups (LOP only, SFL, SFM and SFH), all four markers decreased monotonically as the SF dose increased, and this trend was statistically significant for each marker (Jonckheere–Terpstra trend test: TNF-α, *p* = 1.6 × 10^−5^; IL-6, *p* = 8.7 × 10^−5^; IL-1β, *p* = 3.3 × 10^−5^; MPO, *p* = 3.2 × 10^−4^). Because these measurements were obtained from 3–4 animals per group, they are interpreted as evidence of a dose-related trend rather than as a quantitative dose–response model.

### 2.4. Effect of SF on Colonic MAPK Signaling

**Figure 4:**
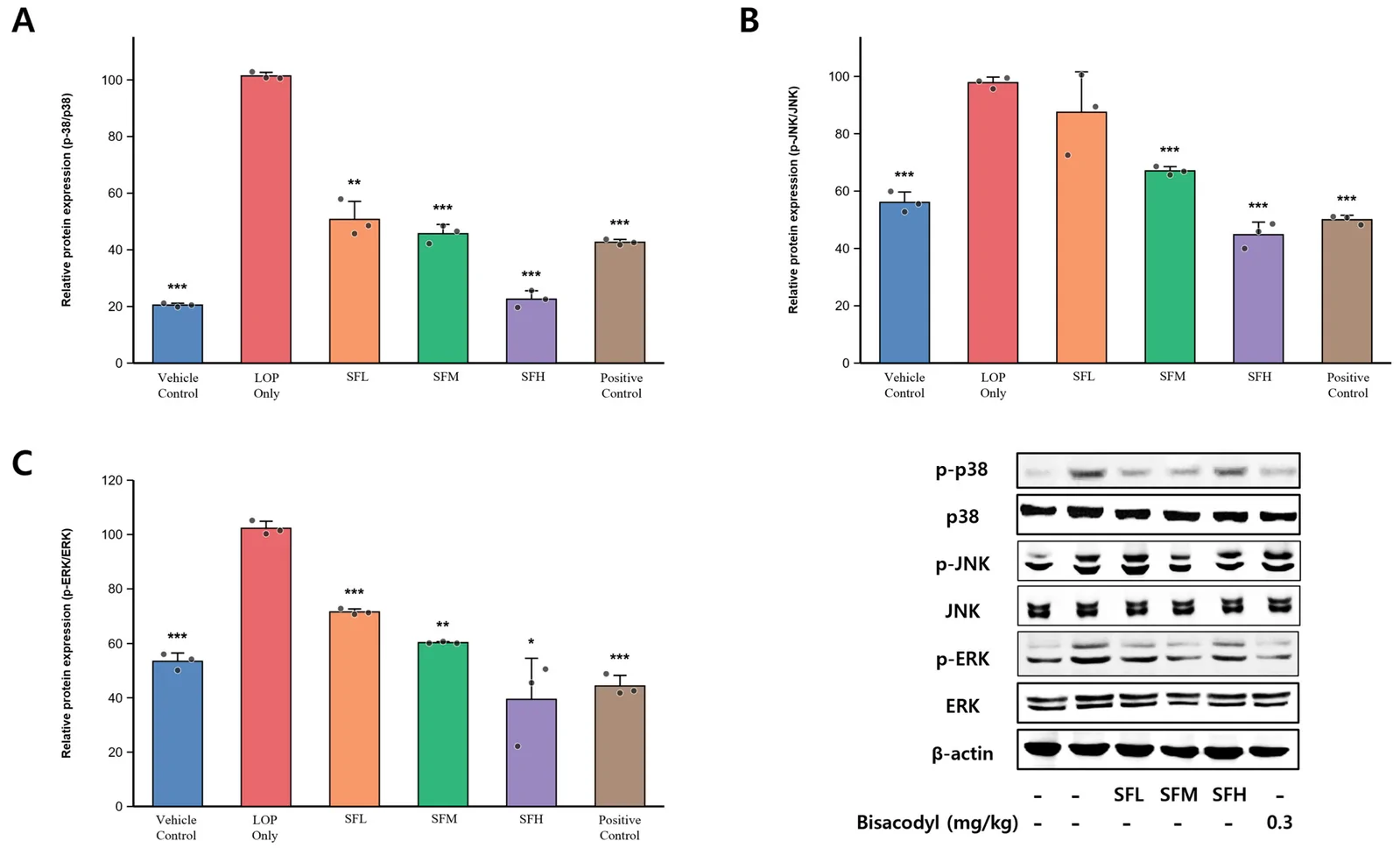
Effect of SF on colonic MAPK signaling in loperamide-induced constipated mice. Relative protein expression of (**A**) phosphorylated p38 (p-p38/p38), (**B**) phosphorylated JNK (p-JNK/JNK), and (**C**) phosphorylated ERK (p-ERK/ERK) in colon tissue, quantified by Western blot. Data are presented as individual values with mean (*n* = 3 per group). * *p* < 0.05, ** *p* < 0.01, *** *p* < 0.001 vs. LOP only.

To elucidate the molecular basis of the observed anti-inflammatory effects, we assessed the phosphorylation status of the three major MAPK proteins in colon tissue (Figure 4). LOP administration markedly elevated p-p38/p38 in the LOP only group compared to Vehicle control (101.39 ± 1.27 vs. 20.48 ± 0.64; *p* < 0.001; Figure 4A). SF dose-dependently attenuated p-p38 phosphorylation: SFL 50.72 ± 6.37 (*p* < 0.01), SFM 45.72 ± 3.23 (*p* < 0.001), and SFH 22.59 ± 2.96 (~78% reduction, approaching the Vehicle control level; *p* < 0.001 vs. LOP only), comparable to the Positive control group (42.67 ± 0.99). p-JNK/JNK was similarly elevated in the LOP only group relative to Vehicle control (97.82 ± 1.94 vs. 56.08 ± 3.59; *p* < 0.001; Figure 4B). SF progressively reduced p-JNK: SFL 87.52 ± 14.10 (not significant), SFM 67.05 ± 1.47 (*p* < 0.001), and SFH 44.81 ± 4.38 (~54% reduction; *p* < 0.001 vs. LOP only), comparable to bisacodyl (50.57 ± 1.16). Likewise, p-ERK/ERK was elevated in the LOP only group (102.31 ± 2.58 vs. 53.45 ± 3.04 in Vehicle control; *p* < 0.001; Figure 4C) and was suppressed by SFL (71.54 ± 1.07; *p* < 0.001), SFM (60.34 ± 0.34; *p* < 0.01), and SFH (39.42 ± 15.15; ~61% reduction; *p* < 0.05 vs. LOP only). These findings indicate that SF attenuates LOP-induced colonic inflammation through dose-dependent inhibition of the p38, JNK, and ERK MAPK signaling pathways.

### 2.5. Effect of SF on Gut Motility-Related Proteins

**Figure 5:**
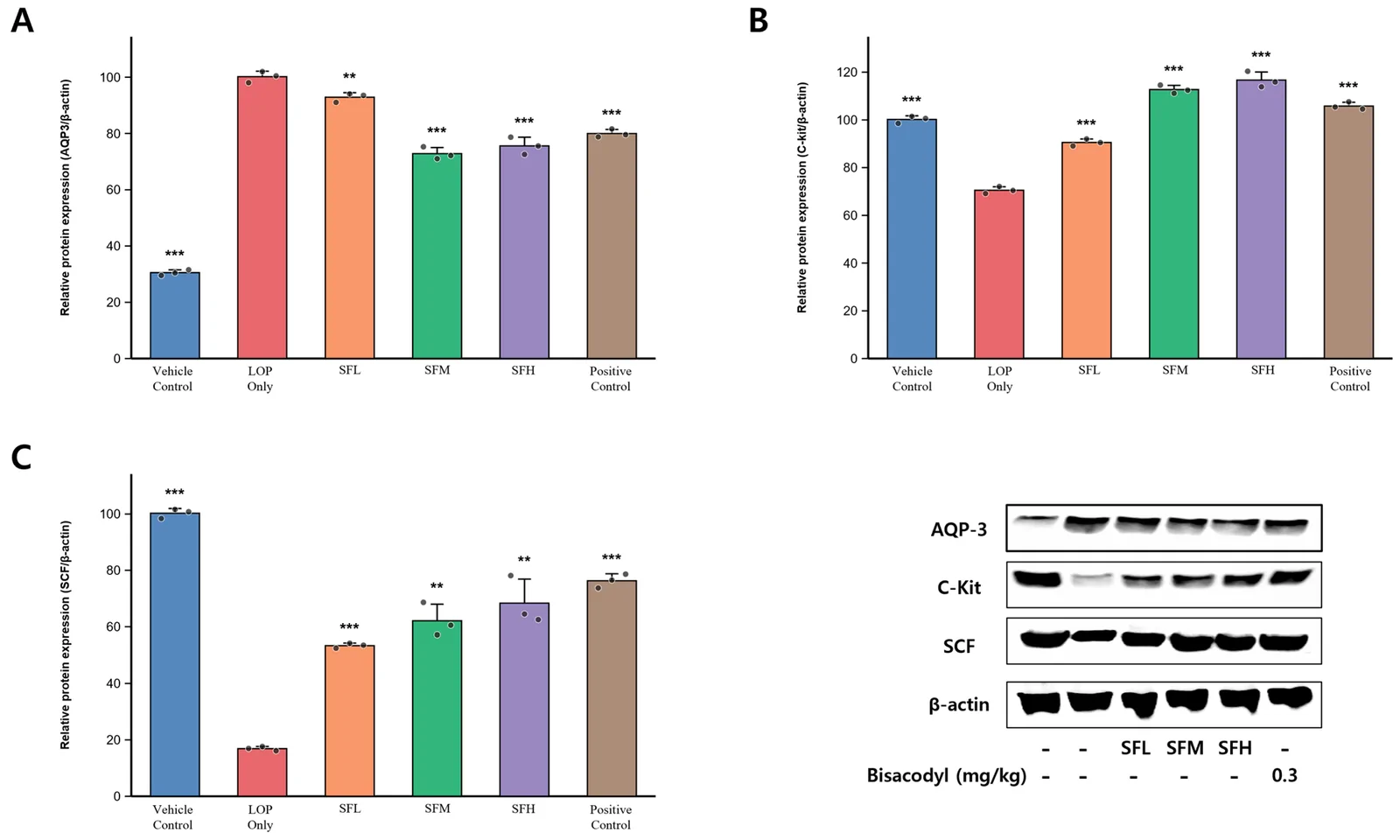
Effect of SF on gut motility-related proteins in loperamide-induced constipated mice. Relative protein expression of (**A**) aquaporin-3 (AQP3/β-actin), (**B**) C-kit (C-kit/β-actin), and (**C**) stem cell factor (SCF/β-actin) in colon tissue, quantified by Western blot. Data are presented as individual values with mean (*n* = 3 per group). ** *p* < 0.01, *** *p* < 0.001 vs. LOP only.

The expression of AQP3, C-kit, and SCF in colon tissue was assessed to evaluate the effect of SF on intestinal motility-related proteins (Figure 5). LOP-induced constipation markedly up-regulated AQP3 expression in the LOP only group relative to Vehicle control (100.15 ± 1.97 vs. 30.51 ± 1.02; *p* < 0.001; Figure 5A). SF dose-dependently limited the increase in AQP3: SFL 92.88 ± 1.61 (*p* < 0.01 vs. LOP only), SFM 72.80 ± 2.20, and SFH 75.60 ± 3.07 (both *p* < 0.001 vs. LOP only), approaching the Positive control group (79.97 ± 1.45). C-kit expression was significantly down-regulated in the LOP only group compared to Vehicle control (70.55 ± 1.44 vs. 100.19 ± 1.52; *p* < 0.001; Figure 5B). SF dose-dependently preserved C-kit expression: SFL 90.53 ± 1.46, SFM 112.72 ± 1.73, and SFH 116.72 ± 3.35 (all *p* < 0.001 vs. LOP only), with SFM and SFH exceeding the Vehicle control level, comparable to the Positive control group (105.49 ± 1.29). SCF showed the most dramatic reduction in the LOP only group (16.90 ± 0.75 vs. 100.26 ± 1.69 in Vehicle control, a ~83% decrease; *p* < 0.001; Figure 5C). SF progressively preserved SCF expression: SFL 53.42 ± 0.88 (*p* < 0.001), SFM 62.12 ± 5.89 (*p* < 0.01), and SFH 68.40 ± 8.48 (*p* < 0.01 vs. LOP only), approaching but not reaching the Vehicle control level, indicating partial but substantial recovery of ICC pacemaker function.

### 2.6. Effect of SF on Colonic Mucosal Structure and Mucin

**Figure 6:**
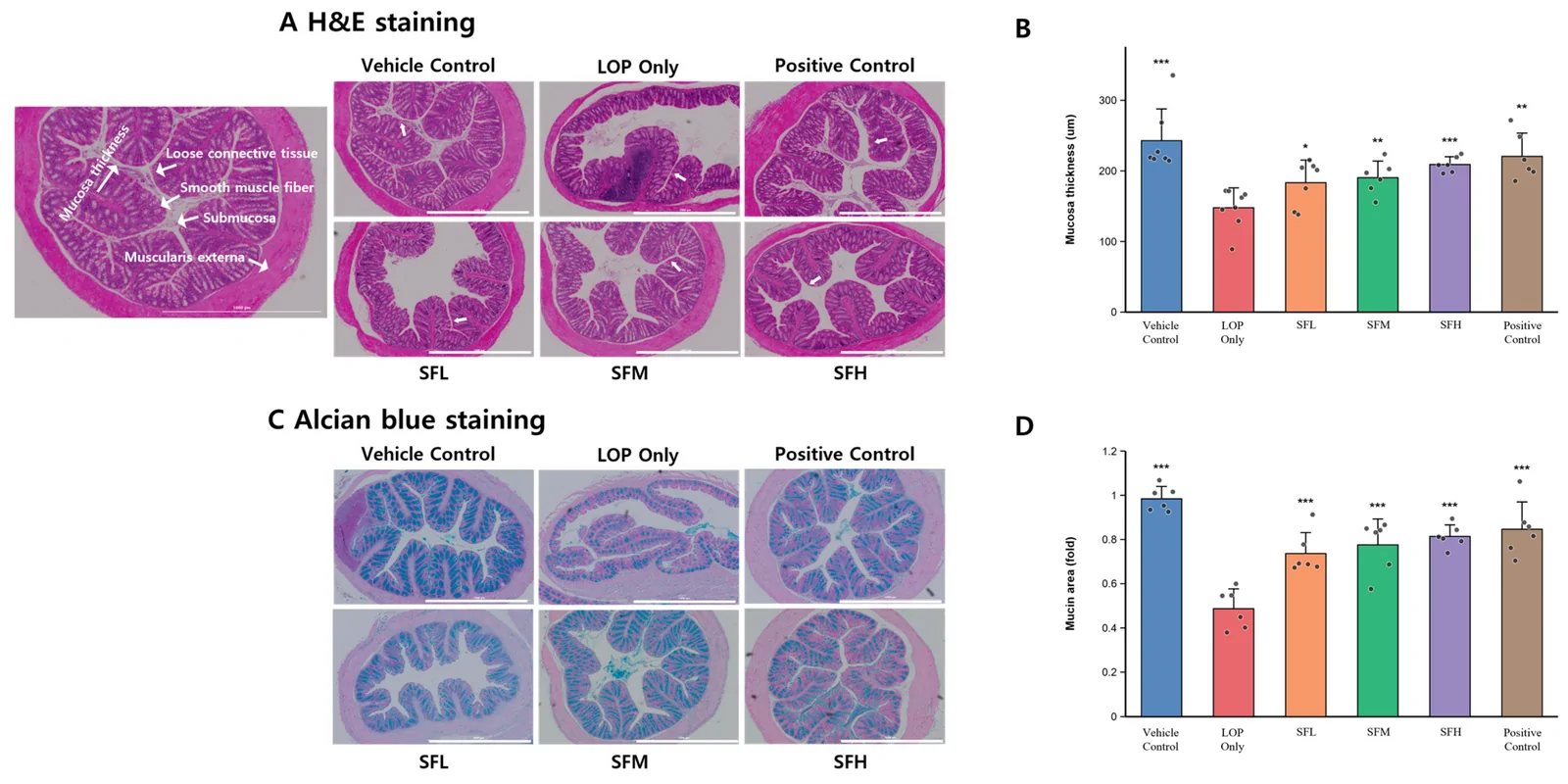
Effect of SF on colonic mucosal structure and mucin in loperamide-induced constipated mice. (**A**) Representative hematoxylin and eosin (H&E)-stained sections of colon tissue. (**B**) Quantification of mucosal layer thickness (µm). (**C**) Representative Alcian blue-stained sections showing mucin-secreting goblet cells. (**D**) Quantification of mucin area expressed as fold change relative to the Vehicle control group. Data are presented as individual values with mean for (**B**) and (**D**); *n* = 6–8 mice per group for (**B**) and *n* = 6 mice per group for (**D**). In (**A**), double-headed arrows indicate the mucosal thickness that was measured. The colonic wall layers are annotated on one representative panel in (**A**): Ep, epithelium; LP, lamina propria (loose connective tissue); MM, muscularis mucosae (smooth muscle fibres); SM, submucosa; ME, muscularis externa. Photomicrographs in (**A**,**C**) were acquired with a ×4 objective, and the scale bars represent 1000 µm. * *p* < 0.05, ** *p* < 0.01, *** *p* < 0.001 vs. LOP only.

Histological analysis using H&E staining (Figure 6A) revealed visible thinning of the colonic mucosa in the LOP only group compared to Vehicle control, which was progressively less marked in the SF-treated groups. Quantification confirmed that mucosal layer thickness was reduced by approximately 40% in the LOP only group relative to Vehicle control (145.26 ± 29.17 vs. 242.84 ± 44.97 µm; *p* < 0.001; Figure 6B). SF dose-dependently preserved mucosal thickness: SFL 183.31 ± 32.12 µm (*p* < 0.05 vs. LOP only), SFM 190.42 ± 23.51 µm (*p* < 0.01 vs. LOP only), and SFH 209.12 ± 11.06 µm (*p* < 0.001 vs. LOP only), approaching the Positive control group (220.60 ± 32.93 µm). Alcian blue staining (Figure 6C) demonstrated a marked reduction in mucin-producing goblet cells in the LOP only group, which was visibly recovered in a dose-dependent manner in the SF-treated groups. Quantification of mucin area (Figure 6D), expressed as fold change relative to Vehicle control, confirmed a significant reduction in the LOP only group (0.49 ± 0.09 vs. 0.98 ± 0.06 in Vehicle control; *p* < 0.001). All SF doses significantly preserved mucin content: SFL 0.74 ± 0.09, SFM 0.78 ± 0.12, and SFH 0.81 ± 0.05 (all *p* < 0.001 vs. LOP only), with SFH comparable to the Positive control group (0.85 ± 0.12).

### 2.7. Effect of SF on the Gut Microbiota

**Figure 7:**
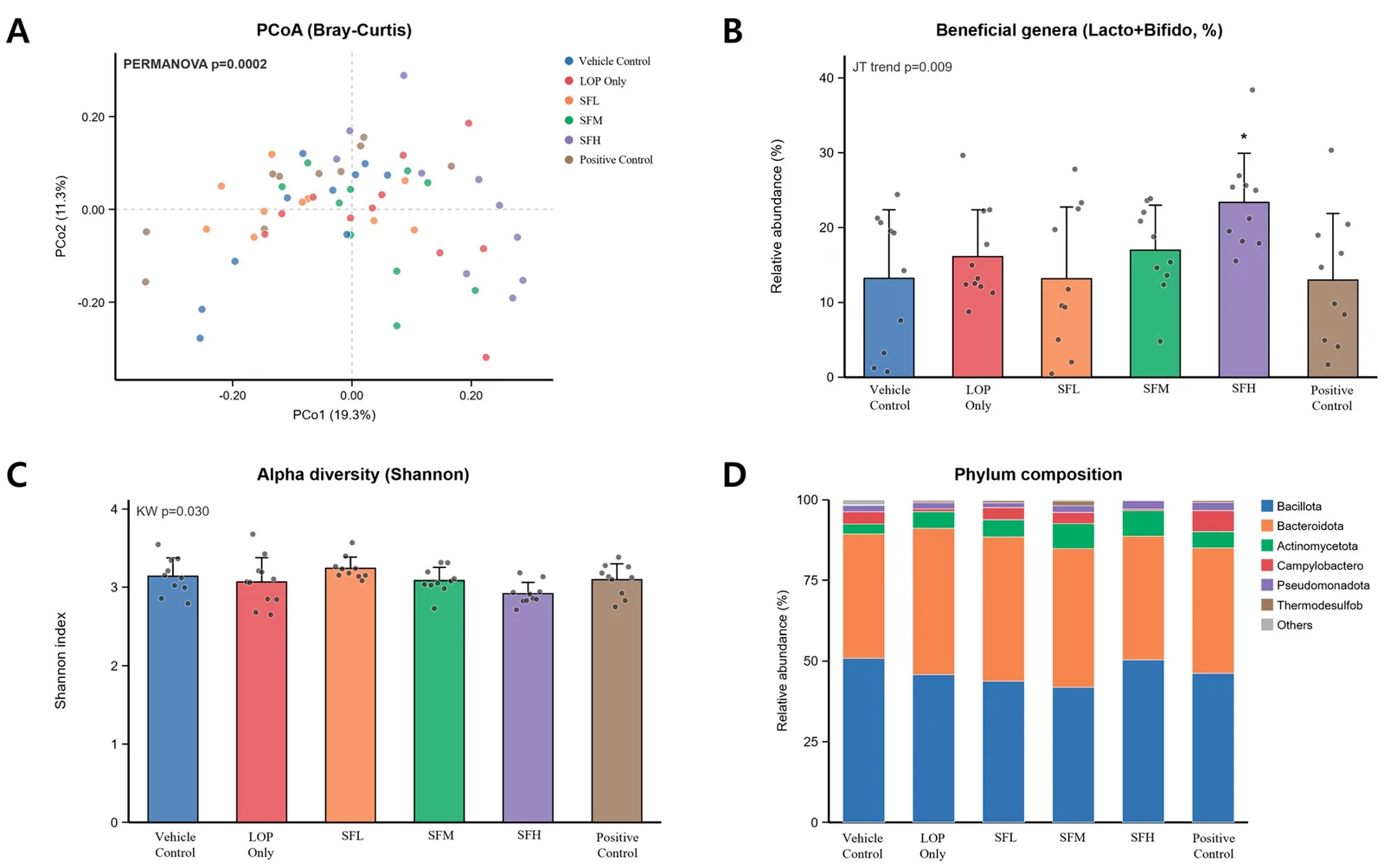
Effect of SF on gut microbiota composition and diversity in loperamide-induced constipated mice. (**A**) Principal coordinates analysis (PCoA) of Bray–Curtis dissimilarity at the genus level. Overall community structure differed significantly among groups (PERMANOVA, *p* = 0.0002; homogeneous dispersion confirmed by PERMDISP, *p* = 0.831). (**B**) Combined relative abundance (%) of the beneficial genera *Lactobacillus* and *Bifidobacterium* across groups (Jonckheere–Terpstra trend test, *p* = 0.009). (**C**) Alpha diversity assessed by the Shannon index (Kruskal–Wallis test, *p* = 0.030; not significant after Benjamini–Hochberg FDR correction). (**D**) Phylum-level microbial composition. Data are presented as individual values with mean for (**B**,**C**) (*n* = 10–11 per group). * *p* < 0.05 vs. LOP only.

PCoA of Bray–Curtis dissimilarity at the genus level revealed a significant separation of community structure among groups (PERMANOVA, overall *p* = 0.0002; Figure 7A). Pairwise comparisons confirmed that all three SF doses differed significantly from the LOP only group (FDR-adjusted q = 0.012, 0.012, and 0.033 for SFL, SFM, and SFH, respectively). Homogeneity of multivariate dispersion was confirmed by PERMDISP (*p* = 0.831), indicating a genuine shift in community centroid rather than a change in within-group variance. The combined relative abundance of the beneficial genera Lactobacillus and Bifidobacterium increased dose-dependently with SF treatment (Figure 7B): LOP only 16.1%, SFL 13.2%, SFM 17.0%, and SFH 23.4% (Jonckheere–Terpstra trend *p* = 0.009), with SFH significantly higher than LOP only (*p* = 0.014). Alpha diversity, assessed by the Shannon index (Figure 7C), was maintained across all groups (Vehicle control 3.14, LOP only 3.07, SFL 3.24, SFM 3.08, SFH 2.92; Kruskal–Wallis *p* = 0.030, not significant after FDR correction), indicating that SF did not reduce overall microbial richness or evenness. At the phylum level (Figure 7D), the microbiota was dominated by Bacillota and Bacteroidota across all groups, with no marked inter-group difference in phylum-level composition.

## 3. Discussion

In the present study, administration of Sargassum fusiforme-derived fucoidan (SF) before and during loperamide exposure was associated with a milder constipated phenotype, together with differences in colonic inflammation, intestinal motility, mucosal integrity, and gut microbiota composition. SF supplementation limited the loperamide-induced decline in fecal output and moisture in a dose-related manner; at 200 mg/kg the relative fecal number and moisture content approached those of the Vehicle control group, although the absolute daily pellet count remained lower than in the Vehicle control and Positive control groups.

The increase in fecal number and moisture could reflect the water-holding properties typical of soluble dietary fibers, an effect on colonic water handling, or both; because the water-holding capacity of the SF preparation was not measured in this study, this remains a hypothesis rather than a demonstrated mechanism. Loperamide suppresses intestinal motility through μ-opioid receptor activation, promoting excessive colonic water reabsorption and producing dry, infrequent stools [39]. Similar improvements in fecal parameters have been reported with diverse natural polysaccharides in LOP-induced constipation models, including Bacillus coagulans, plant extracts, and dietary fibers [40,41]. Notably, the efficacy of SFH across fecal number, relative fecal number, and moisture was comparable to bisacodyl, suggesting that SF achieves its laxative effects through the modulation of motility-regulatory pathways, as evidenced by the downstream findings.

A central finding of this study was the dose-dependent attenuation of MAPK signaling (p-p38, p-JNK, p-ERK) alongside suppression of pro-inflammatory cytokines (TNF-α, IL-6, IL-1β, MPO). These observations are mechanistically linked, as the p38 and JNK pathways are primary upstream regulators of pro-inflammatory cytokine transcription in intestinal tissue, sustaining a self-amplifying inflammatory cycle that impairs both colonic motility and mucosal integrity [19,20]. Highly sulfated fucoidans have been reported to suppress LPS-induced pro-inflammatory cytokine production [30], and fucoidan bioactivity is strongly structure-dependent, with the degree of sulfation among the principal determinants [29]. This is further supported by previous structural and mechanistic characterization of SF-derived fucoidan, in which highly sulfated fractions showed the strongest suppression of LPS-induced pro-inflammatory cytokine production and were predicted to inhibit TLR4-MD2 complex dimerization, an upstream event in MAPK/NF-κB activation [42]. Whether such an upstream mechanism underlies the changes in MAPK phosphorylation observed here was not tested and remains to be determined. SF administration was also associated with higher expression of the ICC pacemaker markers C-kit and SCF, which were severely depleted by LOP—SCF fell to only ~17% of the Vehicle control level in the LOP only group. Loss of ICC, reflected by decreased C-kit expression, is a recognized feature of slow-transit constipation that impairs the generation and propagation of colonic slow waves [43]. SF also normalized the elevated AQP3, a colonic water channel whose over-expression drives excessive luminal water reabsorption and fecal dehydration [23,44]. The parallel differences in ICC marker and AQP3 expression are consistent with, but do not establish, a mechanistic basis for the differences observed in fecal output and moisture.

SF dose-dependently preserved colonic mucosal thickness and mucin content. Mucins, secreted by goblet cells, form the protective mucus layer that maintains barrier integrity and lubricates intestinal transit; their depletion exposes the epithelium to luminal bacteria, amplifying inflammation and perpetuating the constipatory phenotype [25,45]. Restoration of mucin secretion following SF treatment mirrors findings with other natural polysaccharides and probiotics in LOP models [46,47]. Lower colonic cytokine concentrations and better preserved mucin content were observed together in the SF-treated groups. Because goblet cell number and turnover were not measured, the relationship between these two observations cannot be resolved from the present data.

SF also modulated gut microbiota composition, with a significant shift in community structure (PERMANOVA *p* = 0.0002) and a dose-dependent enrichment of Lactobacillus and Bifidobacterium (LOP only: 16.1% → SFH: 23.4%). As a non-digestible sulfated polysaccharide, SF serves as a fermentable substrate that selectively supports the growth of these saccharolytic beneficial genera—a prebiotic effect consistent with multiple in vitro fermentation studies demonstrating fucoidan-driven enrichment of Bifidobacterium and Lactobacillus alongside increased short-chain fatty acid production [31,34]. These beneficial bacteria, in turn, promote colonic motility through serotonin-mediated pathways and support mucin secretion via SCFA signaling, reinforcing the multi-target effects of SF observed across the other endpoints [46]. Notably, alpha diversity was maintained across all groups, indicating that SF selectively restructured community composition without disrupting overall microbial richness.

Taken together, the changes observed across these endpoints are mutually consistent: lower MAPK phosphorylation accompanied lower colonic cytokine concentrations; higher C-kit and SCF accompanied greater fecal output; AQP3 did not rise to the extent seen in the loperamide-only group; mucin content was better preserved; and the relative abundance of Lactobacillus and Bifidobacterium increased with dose. Because these endpoints were measured in parallel rather than manipulated independently, the present data establish association rather than a causal chain, and the individual mechanisms proposed above remain to be tested directly.

Limitations. Several limitations should be considered when interpreting these findings. First, only male ICR mice were studied, so the results cannot be generalized to females. Second, the design was preventive rather than therapeutic: SF was administered for 28 days before loperamide exposure, so the data do not address the treatment of established constipation. Third, no SF-only group was included; the effects of SF in non-constipated animals, and any laxative or other effects at baseline, therefore cannot be assessed from this design. Fourth, fecal pellets were collected from trays beneath group-housed cages rather than from individual animals, so the cage (*n* = 3 cages per group, each housing 3–4 mice) is the experimental unit for the defecation endpoints and the reported variability is between-cage rather than between-animal. Fifth, because the colon of each animal was divided among histological, protein and cytokine analyses, the ELISA measurements (*n* = 3–4 per group) and the Western blot analyses (*n* = 3 per group) were performed on a subset of animals; although the dose-related trend in the cytokine and MPO data was statistically significant, these analyses were not powered for quantitative dose–response modelling and should be regarded as supporting rather than primary evidence. Sixth, the material tested was a standardized extract of *S. fusiforme* rather than a purified single polysaccharide, so the effects observed cannot be attributed to fucoidan alone. Seventh, the water-holding capacity of the preparation, intestinal permeability, bacterial translocation and goblet cell turnover were not measured, so the mechanistic interpretations offered above remain hypotheses. Finally, the gut microbiota were characterized by 16S rRNA gene amplicon sequencing, which does not resolve function; no short-chain fatty acid or metabolomic measurements were made, and no-template PCR controls or extraction blanks were not processed alongside the samples, so low-level reagent contamination cannot be formally excluded. Confirmatory studies with adequate group sizes, both sexes, a therapeutic design and complete material characterization will be required before the utility of SF in the management of constipation can be assessed.

## 4. Materials and Methods

### 4.1. Chemicals

Loperamide hydrochloride (LOP) and bisacodyl were purchased from Sigma-Aldrich (St. Louis, MO, USA). The fucoidan used in this study was manufactured by S&D Co., Ltd. (Cheongju, Republic of Korea) through a standardized extraction and purification process from dried *Sargassum fusiforme* (Lot.SD-SF-001). Briefly, the dried seaweed was subjected to alkaline extraction at 75 °C for 2 h, followed by enzymatic hydrolysis (pH 4.5, 50 °C, 24 h). After heat-inactivation at 95 °C, the extract was filtered through a 25 μm membrane, treated with calcium chloride to remove alginate, and concentrated via ultrafiltration (3 kDa cut-off). The final product was sterilized at 85 °C and spray-dried to yield a standardized powder. The chemical composition of SF was determined as follows: total carbohydrate content, 50.30 ± 0.45%; fucose content, 9.6 ± 0.1%; and sulfate group content, 29.10 ± 0.18%, confirming its identity as a highly sulfated fucoidan derived from *S. fusiforme.* The monosaccharide composition of the lot used here was determined in-house by reversed-phase HPLC with ultraviolet detection following acid hydrolysis and pre-column derivatization with 1-phenyl-3-methyl-5-pyrazolone (PMP), and was fucose 9.60 ± 0.10%, galactose 6.05 ± 0.18%, xylose 2.78 ± 0.09% and mannose 1.73 ± 0.06% (*w*/*w* of dry powder); glucose, guluronic acid and mannuronic acid were not detected, indicating that neither laminaran- nor alginate-derived material was carried through the purification. The fucose-to-galactose ratio of approximately 1.6:1, together with the sulfate content, is consistent with a sulfated fucogalactan. The chromatogram and the full composition are provided as Supplementary Figure S1. All other reagents and chemicals used in this study were of analytical grade.

### 4.2. Animals and Experimental Design

Seven-week-old male ICR mice were purchased from a certified supplier and housed in a specific pathogen-free facility under a 12 h light/dark cycle at 23 ± 2 °C and 50 ± 10% relative humidity, with standard laboratory chow and water provided ad libitum. Following a 10-day acclimatization period (Day −10 to Day 0), all animals were weighed and ranked by body weight and were then allocated to the six groups so that animals of similar body weight were distributed evenly among them. This weight-matched allocation ensured that mean initial body weight did not differ among groups at the start of the experiment. We note that a formal randomization sequence was not generated, and the text has been corrected accordingly: the original description of the animals as “randomly assigned” has been replaced by this account of what was actually done. The final group sizes were Vehicle control *n* = 10, LOP only *n* = 11, Positive control *n* = 10, SFL *n* = 10, SFM *n* = 10 and SFH *n* = 10 (61 animals in total); one additional animal was allocated to the LOP only group at the outset to allow for possible mortality during loperamide administration, but no mortality occurred in any group and no animal was excluded from analysis. The groups were: (1) Vehicle control group: vehicle only; (2) LOP only group: loperamide hydrochloride (5 mg/kg); (3) Positive control group: loperamide + bisacodyl (0.3 mg/kg); (4) low-dose group (SFL): loperamide + SF 50 mg/kg; (5) medium-dose group (SFM): loperamide + SF 100 mg/kg; (6) high-dose group (SFH): loperamide + SF 200 mg/kg. All animal procedures were conducted in accordance with the guidelines for the care and use of laboratory animals and were approved by the Animal Experimental Ethics Committee of Jeju National University (approval number: 2022-0005).

### 4.3. Administration and Induction of Constipation

SF and bisacodyl were dissolved in sterile distilled water and administered by daily oral gavage for 35 consecutive days (Day 1–35) at a fixed volume of 10 mL/kg body weight, adjusted to the body weight recorded at the most recent weighing. All administrations were performed once daily at the same time of day during the light phase (lights on at 06:00 and off at 18:00). The Vehicle control group received an equivalent volume of vehicle throughout. To induce constipation, loperamide hydrochloride (5 mg/kg) was administered by oral gavage once daily during the final 7 days of the experiment (Day 29–35). The Vehicle control group was not subjected to loperamide treatment. This model is well-established for inducing constipation in rodents through opioid receptor-mediated inhibition of intestinal motility [39].

### 4.4. Measurement of Growth and Physiological Parameters

Body weight was recorded weekly during the SF administration period (Weeks 1–5) and daily during the loperamide induction period (Days 29–35). Relative body weight was calculated by normalizing each daily measurement to the body weight recorded on Day 29 (first day of loperamide administration). Feed intake and water intake were measured daily from Day 29 to Day 35 by subtracting the residual amount from the quantity supplied on the previous day.

### 4.5. Evaluation of Fecal Parameters

To assess bowel movement, fecal pellets were collected daily from Day 29 to Day 35. Plastic bottom trays lined with absorbent pads were placed beneath wire-mesh cage floors to allow feces to fall through and be separated from bedding. Mice were group-housed in 3 cages per group (3–4 mice per cage). Pellets recovered from each tray were counted and divided by the number of mice housed in that cage to give a per-animal daily pellet count for that cage. Individual animals could not be identified for this endpoint, so the cage—not the mouse—is the experimental unit, and all statistical comparisons for fecal number and relative fecal number were performed on cage-level values (*n* = 3 cages per group). Relative fecal number was calculated by normalizing daily counts to the value recorded on Day 29 (baseline = 1.0). Fecal pellets were collected once daily at the same time of day, immediately before dosing and cage change, so that each daily count corresponds to a consistent 24-h collection interval.

### 4.6. Measurement of Fecal Water Content

Fecal water content was measured on Day 35. Each mouse was individually placed in a clean cage for 30 min, and fecal pellets spontaneously excreted during this period were immediately collected. Pellets were weighed (wet weight), dried at 105 °C to constant weight (dry weight), and fecal moisture content (%) was calculated as [(wet weight − dry weight)/wet weight] × 100.

### 4.7. Necropsy and Tissue Collection

At the end of the experimental period (Day 35), and following the behavioral assessment, mice were euthanized by CO_2_ inhalation without prior anesthesia, in accordance with the protocol approved by the Institutional Animal Care and Use Committee of Jeju National University (2022-0005), and necropsy was performed immediately. The entire colon was carefully excised from the cecum to the anus, and colon length was measured using a precision ruler. Colon tissues were divided into three portions: one segment was fixed in 4% paraformaldehyde (PBS, pH 7.4) for histological analysis; a second segment was snap-frozen in liquid nitrogen and stored at −80 °C for protein extraction; and a third segment was stored at −80 °C for cytokine and enzyme measurement. Fecal contents were collected from the cecum and stored at −80 °C for microbiota analysis.

### 4.8. Measurement of Colonic Pro-Inflammatory Cytokines and MPO

Colonic tissue (50 mg) was homogenized in ice-cold 1× PBS using a tissue homogenizer, subjected to two freeze–thaw cycles to lyse cells completely, and centrifuged at 10,000× *g* for 15 min at 4 °C. Total protein concentration in each supernatant was determined using the Pierce BCA Protein Assay Kit, and all supernatants were adjusted to the same total protein concentration before assay so that the values reported for different samples are directly comparable. The resulting supernatants were used to quantify TNF-α, IL-6, IL-1β, and myeloperoxidase (MPO) by enzyme-linked immunosorbent assay (ELISA) using commercially available kits (MyBioSource, San Diego, CA, USA) according to the manufacturers’ protocols. ELISA kits were as follows: TNF-α, Cat. MBS825075; IL-1β, Cat. MBS175967; IL-6, Cat. MBS2023471; myeloperoxidase, Cat. MBS700747 (all MyBioSource, San Diego, CA, USA). All assays were colorimetric and absorbance was read at 450 nm; the assay range and sensitivity of each kit are as specified by the manufacturer. All samples were assayed in duplicate and concentrations were interpolated from a four-parameter logistic standard curve. Because the colon of each animal was divided among histological analysis, protein extraction and cytokine measurement, sufficient supernatant for the complete four-analyte ELISA panel was available from 3–4 animals per group, and the ELISA measurements were therefore performed on that subset; animals were not selected on the basis of their outcome values, and every individual value obtained is shown in Figure 3.

### 4.9. Western Blot Analysis

Colon tissues were lysed in RIPA buffer (Thermo Fisher Scientific, Waltham, MA, USA) supplemented with protease and phosphatase inhibitor cocktails (GenDEPOT, Katy, TX, USA). Lysates were centrifuged at 14,000 rpm for 20 min at 4 °C, and protein concentrations were determined using the Pierce BCA Protein Assay Kit (Thermo Fisher Scientific, Waltham, MA, USA). Protein concentration was determined by BCA assay and 20 µg of protein per lane was resolved by 10–12% SDS-PAGE, transferred onto nitrocellulose membranes (Amersham, 0.45 μm; Cytiva, Freiburg, Germany), and blocked with 5% bovine serum albumin in TBST for 2 h at room temperature. Membranes were incubated overnight at 4 °C with primary antibodies against phospho-p38, total p38, phospho-JNK, total JNK, phospho-ERK, total ERK (Santa Cruz Biotechnology, Dallas, TX, USA), AQP3, C-kit, SCF, and β-actin (all 1:1000 dilution). After washing, membranes were incubated with horseradish peroxidase-conjugated secondary antibodies (1:10,000) for 2 h at room temperature. Bands were detected using an enhanced chemiluminescence substrate (Amersham) and quantified with ImageJ software (version 1.54p; NIH, Bethesda, MD, USA). Phosphorylation levels were expressed as ratios of phosphorylated to total protein; AQP3, C-kit, and SCF were normalized to β-actin. For each target, samples from all six groups (*n* = 3 per group) were resolved on the same gel and transferred to the same membrane, so that all groups shown in a given panel are directly comparable; phosphorylated and total forms of each MAPK were detected on separate membranes run in parallel, and the complete uncropped images of every membrane are provided as Supplementary Material. β-actin was detected on the same membrane as the corresponding target for every gel, and band intensities were comparable across all lanes, so that loading can be assessed directly from the uncropped images. All values are expressed relative to the LOP only group (set to 100; *n* = 3 per group). Full uncropped blot images for all targets are provided as Supplementary Material.

### 4.10. Histological Analysis of Colonic Mucosa and Mucin

Paraformaldehyde-fixed, paraffin-embedded colon tissues were sectioned at 5 μm thickness. After deparaffinization and rehydration, sections were stained with hematoxylin and eosin (H&E) to evaluate mucosal layer thickness and overall histomorphology. Mucin content was assessed by Alcian Blue/Periodic Acid–Schiff (AB/PAS) staining. Digital images were acquired using a Carl Zeiss confocal microscope (Oberkochen, Germany) and analyzed with ImageJ software. For histological analysis, a segment of approximately 1 cm of distal colon was taken beginning 1 cm proximal to the anus. Segments were embedded as transverse rings, and three non-serial sections per animal were examined. Mucosal thickness was measured across the entire length of each section rather than at a selected subset of sites, and the measurements were averaged to give a single value per animal, so that *n* refers to the number of animals. Mucin area was quantified and expressed as fold change relative to the Vehicle control group (*n* = 6–8 per group).

### 4.11. Gut Microbiota Analysis

Fecal DNA was extracted using the DNeasy PowerSoil Pro Kit (Qiagen, Hilden, Germany). DNA quality and quantity were assessed with a NanoDrop One spectrophotometer and a Qubit Flex Fluorometer (Thermo Fisher Scientific). Libraries were prepared following the 16S Metagenomic Sequencing Library Preparation protocol (Illumina). The V3–V4 hypervariable region of the 16S rRNA gene was amplified with the primer pair of Klindworth et al. (341F, 5′-CCTACGGGNGGCWGCAG-3′; 805R, 5′-GACTACHVGGGTATCTAATCC-3′), each carrying the Illumina overhang adapter sequence, using KAPA HiFi HotStart ReadyMix under the following conditions: 95 °C for 3 min; 25 cycles of 95 °C for 30 s, 55 °C for 30 s and 72 °C for 30 s; and a final extension at 72 °C for 5 min. Amplicons were purified with AMPure XP beads, indexed by limited-cycle PCR using the Nextera XT Index Kit (Illumina, Inc., San Diego, CA, USA), purified again, quantified fluorometrically, pooled in equimolar amounts and sequenced on the Illumina MiSeq platform (Illumina, San Diego, CA, USA). Bioinformatic processing of the sequencing data was performed by the service provider. Reads were processed in QIIME2, including primer trimming, denoising, merging and chimera filtering, and taxonomy was assigned to the genus level; full details of the provider’s pipeline, including the denoising algorithm, its truncation parameters and the version of the reference taxonomy database, are available in the provider’s processing report on request. Because the taxonomic table available for the present analysis was supplied at collapsed taxonomic levels, no ASV-level analyses were performed. Mitochondrial, chloroplast and unclassified reads were removed. Sequencing depth ranged from 250,460 to 1,749,501 reads per sample (median 568,562). No-template PCR controls and reagent-only extraction blanks were not included in this study; because fecal samples are high-biomass, low-level reagent contamination is unlikely to have affected the community profiles materially, but it cannot be formally excluded. Relative abundances were calculated for each sample as the read count for a given taxon divided by the total number of reads for that sample, and beta-diversity analyses were performed on these compositional data without rarefaction. Because alpha-diversity indices are sensitive to sequencing depth, alpha diversity was instead computed after rarefying each sample to the lowest observed depth (250,460 reads), averaged over 100 independent rarefactions. Rarefaction did not materially change the values or the outcome of the statistical tests. Beta-diversity was assessed by principal coordinates analysis (PCoA) of Bray–Curtis dissimilarity at the genus level; community-level differences were tested by PERMANOVA (4999 permutations) and verified by PERMDISP. Alpha diversity was evaluated using the Shannon index (Kruskal–Wallis test with Benjamini–Hochberg FDR correction). The dose-dependent trend in the combined relative abundance of Lactobacillus and Bifidobacterium was evaluated by the Jonckheere–Terpstra trend test (*n* = 10–11 per group).

### 4.12. Statistical Analysis

All data are expressed as mean ± standard deviation (SD). Pairwise comparisons between the LOP only group and each treatment group were performed using Welch’s *t*-test or the Mann–Whitney U test, as appropriate following normality assessment. Statistical significance was defined as *p* < 0.05. Dose-related trends across the ordered groups (LOP only, SFL, SFM, SFH) were evaluated using the Jonckheere–Terpstra trend test for the colonic cytokine and MPO data. For microbiota analyses, the Kruskal–Wallis test, PERMANOVA (4999 permutations), the Jonckheere–Terpstra trend test, and Benjamini–Hochberg FDR correction were applied as described in Section 4.11. All analyses were performed using GraphPad Prism (version 10.0; GraphPad Software, San Diego, CA, USA) and R software (version 4.3; R Foundation for Statistical Computing, Vienna, Austria). Significance levels are indicated as * *p* < 0.05, ** *p* < 0.01, and *** *p* < 0.001 vs. LOP only.

## 5. Conclusions

In male ICR mice, administration of *Sargassum fusiforme*-derived fucoidan for 35 days before and during loperamide exposure effectively reduced the constipated phenotype, increasing fecal output and fecal moisture content relative to loperamide-treated controls in a dose-related manner. These changes were accompanied by lower colonic pro-inflammatory cytokine and MPO concentrations, which decreased monotonically with dose, by reduced MAPK phosphorylation, by altered expression of the motility-related proteins C-kit, SCF and AQP3, by better preserved colonic mucosal architecture and mucin content, and by a shift in fecal microbiota composition with enrichment of Lactobacillus and Bifidobacterium. Because the design was preventive rather than therapeutic and only male animals were studied, confirmation in a therapeutic design and in both sexes will be required before the utility of SF fucoidan in the management of constipation can be established. These findings nevertheless support *S. fusiforme* fucoidan as a promising candidate ingredient for the dietary management of constipation.

## Data Availability

The original contributions presented in this study are included in the article/supplementary material. Further inquiries can be directed to the corresponding author.

## Supplementary Materials

The following supporting information can be downloaded at: https://www.mdpi.com/article/10.3390/md24080266/s1, Figure S1: Monosaccharide composition of the *Sargassum fusiforme* fucoidan preparation (SF) used in this study.

## Abbreviations

The following abbreviations are used in this manuscript:

SF	*Sargassum fusiforme*-derived fucoidan
SFL	*Sargassum fusiforme*-derived fucoidan low-dose group
SFM	*Sargassum fusiforme*-derived fucoidan middle-dose group
SFH	*Sargassum fusiforme*-derived fucoidan high-dose group
LOP	Loperamide
MPO	Myeloperoxidase
MAPK	Mitogen-activated protein kinase
ICC	Interstitial cell of Cajal
SCF	Stem cell factor
AQP3	Aquaporin-3
