# Supplementary material for: A Sargassum fusiforme-Derived Fucoidan Preparation Ameliorates Loperamide-Induced Constipation and Is Associated with Changes in Colonic Inflammation and Gut Microbiota in Mice
Source: Mar Drugs. 2026 Jul 31;24(8):266. doi: 10.3390/md24080266 (PMC13514422; doi:10.3390/md24080266)
Supplement: Supplementary file 1 [file marinedrugs-24-00266-s001.zip › marinedrugs-4468012-supplementary.pdf]

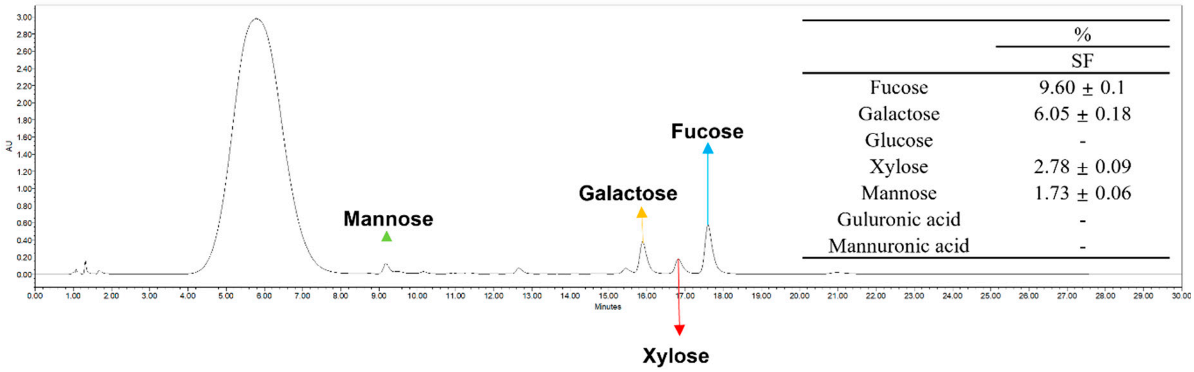

	%
	SF
Fucose	9.60 ± 0.1
Galactose	6.05 ± 0.18
Glucose	-
Xylose	2.78 ± 0.09
Mannose	1.73 ± 0.06
Guluronic acid	-
Mannuronic acid	-

Figure S1. Monosaccharide composition of the *Sargassum fu-siforme* fucoidan preparation (SF) used in this study.

The sample (10 mg) was hydrolyzed with 1 mL of 2 M TFA at 100 °C for 2 h. After cooling, the hydrolysate was filtered, neutralized with 2 M NaOH, and diluted with distilled water. For PMP derivatization, 2 mL of the hydrolysate was mixed with 2 mL each of 0.5 M PMP in methanol and 0.3 M NaOH, followed by incubation at 70 °C for 30 min. The mixture was neutralized with 2 mL of 0.3 M HCl and extracted with 4 mL of chloroform. After centrifugation at 1,500 rpm for 15 min, the aqueous phase was filtered through a 0.45 μm PTFE filter and subjected to HPLC analysis.
